# Specific cultural traits of the precarity of older Latinos living alone with cognitive impairment in the San Francisco Bay Area

**DOI:** 10.1080/13607863.2024.2407180

**Published:** 2024-09-27

**Authors:** Daniel Velez Ortiz, Nicole Ransom, Elizabeth Rivera, Julene K. Johnson, Sahru Keiser, Thi Tran, Jacqueline Torres, Elena Portacolone

**Affiliations:** a Michigan State University, East Lansing, MI, USA;; b University of California San Francisco, San Francisco, CA, USA

**Keywords:** Cognitive impairment, living alone, precarity, older Latinos, culture

## Abstract

**Objectives:**

Latinos are about twice as likely to develop cognitive impairment. Culturally, filial support and familismo are expected within Latino families. Yet approximately twenty percent of Latinos live alone in the United States. The purpose of this study is to explore the concerns and priorities of older Latinos living alone with cognitive impairment, using a precarity framework.

**Method:**

We conducted 22 in-home interviews with older Latinos living alone with cognitive impairment, and we supplemented the interviews with interviews with members of the older adults’ social circle and providers. Themes influencing the precarity of Latino older adults living alone were organized through the major areas of the precarity lens; 1) Limited awareness of cognitive impairment; 2) Self-management of cognitive impairment; and 3) Lacking tailored services for cognitive impairment.

**Results:**

Latino culture permeated and intersected across the lived experiences of participants living alone with cognitive impairment. Precarity was prevalent in all participants’ lives and was exacerbated by familismo combined with cognitive impairment.

**Conclusion:**

Findings showed precarity in the experiences the participants shared. Participant narratives reveal how the Latino culture intersects with the experience of precarity while living alone with cognitive impairment, especially in reference to the role that family plays through the expectation of familismo. However, given the difficult demands of employment and raising their own families, familismo can become more like a goal than a practice. Further research is needed to better understand how to bridge the gap between the needs of these older Latino adults living alone with cognitive impairment, their families, and formal services.

## Introduction

Despite the belief that older Latinos (aged 65 and over) live with their families, 20% of them live alone in the United States (US) (U.S. Department of Health and Human Services, Administration for Community Living, 2018). Furthermore, Latinos are twice more likely than Whites to develop cognitive impairment (Gupta, 2021). Thus, a considerable portion of older Latinos who live alone are likely to have cognitive impairment, ranging from mild cognitive impairment to Alzheimer’s disease and related dementias. Previous studies, primarily on older Whites, have shown that older adults with cognitive impairment who live alone, compared to those living with cohabitants, are at high risk for health threats including self-neglect (Gibson & Richardson, 2017; Meaney et al., 2005; Miranda-Castillo et al., 2010; Yu et al., 2021), malnutrition (Miranda-Castillo et al., 2010; Tuokko et al., 1999), and falls (Miranda-Castillo et al., 2010; Tierney, 2007; Tuokko et al., 1999). Further, another study pointed out that about one third of Latinos living alone with cognitive impairment are likely not to receive any help when having difficulties managing activities of daily living, including instrumental activities (Edwards et al., 2020).

Specifically, there is a knowledge gap in understanding how cultural norms may influence the experience of living alone with cognitive impairment in Latino communities (González et al., 2019; Mayeda et al., 2016). To that end, a growing body of cognitive impairment research suggests that risk factors among Latinos are nuanced, thus necessitating further examination of the relationship between cognitive impairment and cultural and acculturation experiences (Gentry et al., 2021; Perales-Puchalt et al., 2021; Rote et al., 2019; Ruiz & Ransford, 2012). For example, one stressor that started emerging in studies by our group is immigration. Specifically, how immigration status can limit a person’s ability to access services due to distrust and fear of authorities relating to the experiences of discrimination and threat of deportation. Another stressor is the Latino cultural value of *familismo* (Ruiz & Ransford, 2012), usually defined as ‘intergenerational solidarity, obligation, respect, and duty to care for one’s own’ (p. 51). In a Latino collectivist culture, family tends to take precedence over individual needs. Thus, there is an expectation, rooted in cultural values and history, that the older adult live with or close to their children and grandchildren and that their caretakers be women in the family (Adames et al., 2014). However, due to shifts in generational expectations, changes in gender roles, and greater geographical dispersion of family members, older Latinos with cognitive impairment, particularly immigrant women, may be unexpectedly living alone with small support networks because their family is no longer easily accessible (Rote et al., 2019; Ruiz & Ransford, 2012). Thus, it is possible that older Latinos living alone with cognitive impairment experience precarity in ways that are culturally unique as living alone is not a cultural norm for them. Living alone may even be stigmatizing because it may point to limited support from family members, which is prized in Latino cultural tradition (Ruiz & Ransford, 2012).

Research by Portacolone et al. (2021) and Portacolone et al. (2019) posited the precarity of older adults living alone with cognitive impairment. Precarity refers to a sense of uncertainty to deal with declines in cognitive, personal, and social resources while trying to dignify living alone. Key findings from the study on precarity present that it manifested in three ways. The first was the difficulty of older adults living alone with cognitive impairment understanding what cognitive impairment is and how it affects them. The second was the difficulty of older adults with cognitive impairment managing tasks related to living alone (Activities of Daily Living and Instrumental Activities of Daily Living) that were complicated by cognitive impairment. Lastly, there was an absence of services to address basic physical and social needs according to their cognitive impairment.

A more recent qualitative study of our group examined how 21 ethnically diverse older adults experienced the COVID-19 pandemic while living alone with cognitive impairment (Portacolone et al., 2019). The precarity of the 8 Latino participants of this study stemmed from having family members outside the US who they could not reach, as well as from living in cramped settings, overly relying on home care aides, and feeling isolated. Apart from this emerging evidence, very little is known about the lived experience of older Latinos with cognitive impairment who are living alone.

Given the previously mentioned factors pointing to Latinos experiencing precarity, the aim of this study is to use the three manifestations of precarity as a framework to guide a deep exploration into the cultural expectations and experiences that may shape the concerns and priorities of older Latinos living alone with cognitive impairment. Next, we will identify whether other themes specifically related to Latino cultures also emerged.

## Design and methods

The data are part of a multiyear qualitative study on living alone with cognitive impairment, The Living Alone with Cognitive Impairment Project. This study uses qualitative research methods to understand, in depth, the subjective experience of living alone with cognitive impairment. Qualitative research studies usually seek to evaluate how individuals interpret their worlds (Creswell, 2013) and the meanings they assign to events. Qualitative interviewing was specifically used in this study because they are designed to elicit an in-depth understanding of the participants’ perspectives. We analyzed the data using deductive and inductive content analysis (Elo & Kyngäs, 2008). The previous findings on precarity served as a framework to study older adults with cognitive impairment living alone, which drives a deductive approach at the outset. We chose to use the previous results as a framework because there is a human experience that threads across those who live alone with cognitive impairment. Thus, the themes contained in the precarity framework would likely be present within the Latino population as well. The inductive approach comes from the cultural context of older Latinos living alone with cognitive impairment. While there is a common human thread across older adults living alone with cognitive impairment, culture provides contextual factors to lived experiences that set them into their own unique themes. The research protocol for ethical conduct and human subjects protection in this study was approved by a university Institutional Review Board.

### Participant eligibility and recruitment

Inclusion criteria were: (a) age ≥55; (b) medical diagnosis of Alzheimer’s disease, mild cognitive impairment or dementia, or a score of ≤24 on the Montreal Cognitive Assessment; (c) self-identifying as Latino or Hispanic; and (d) living alone. Exclusion criterion was residing in institutional settings (e.g. continuum of care facilities, assistive living facilities). To recruit informants, we also asked participants if we could interview one member of their social circles. Finally, to gain further insights, we interviewed Latino providers who served older adults with cognitive impairment and helped us with recruitment. Participant recruitment was based on referrals from administrative staff, physicians, and social workers of health care organizations based in the 9 counties of the Bay Area of San Francisco.

### Data collection

One of the authors developed the qualitative interview protocol, and a co-author reviewed it to ensure that the questions were open-ended and elicited information on different elements of participants’ lived experience about living alone with cognitive impairment. Interviews were conducted by the team, including two Latina researchers. Most interviews occurred in participants’ homes. Participants were asked about their experiences of living alone, their likes/dislikes regarding their living arrangement, and what resources were essential for them. Participants were also asked what they thought about their cognitive function, such as their experience with remembering events and finding the right words during conversations. If they believed that they had difficulty with cognitive function, they were asked about strategies they employed to cope. Researchers also asked participants what informal and formal support networks they used, and their opinions about them (e.g. questions elicited what they liked about them). Data from interviews were supplemented with data from researcher observation of participants’ routines during visits. The researcher’s audio recorded field notes of the participants’ appearance, body language and physical environment. To ensure reflexivity, they carefully recorded their reactions to what was observed and their possible bias and role in the encounters. In this way, the researchers used their experiences in relation to the studied population as part of the data (Creswell, 2013). The interviews were audiotaped and lasted approximately 1.5 h. Home visits lasted approximately 2.5 h. Participants received a $30 gift card at the first interview. Because the participants had mild cognitive impairment, we conducted several interview sessions with each participant to address different parts of the interview protocol. Breaking up the interview protocol questions was more realistic and tolerable to participants.

### Data analysis

We interviewed older Latinos living alone with cognitive impairment, and we supplemented the interviews with interviews with members of the older adults’ social circle and providers. Using precarity as a framework involved two steps. First, we explored if the three themes related to precarity that emerged in prior studies by our group (i.e. awareness of cognitive impairment, self-management of the challenges caused by the cognitive impairment and lacking tailored services) also emerged among Latino participants. Data analysis had several steps. First, drawing from deductive analysis, the first and second authors reviewed the transcripts independently to identify whether the themes that evoked precarity (i.e. awareness of cognitive impairment, self-management of the challenges caused by the cognitive impairment and lacking tailored services) also emerged among Latino participants and the potential cultural overlays of Latino cultures in the manifestation of cognitive impairment while living alone. To reflect precarity, the themes had to have at least 4 markers of precarity embedded: uncertainty, cumulative pressures, limited access to appropriate services and/or wanting to be independent. To better understand the four markers, please see our prior studies (Portacolone et al., 2018, 2019).

Throughout the review of participant interviews came identifying data that represented the precarity themes. Also, there was data that did not fit the precarity themes and were coded as their own category. A senior coder reviewed the codes until they achieved convergence in their interpretations. Definitions of codes and related categories were documented in a code-book and shared with the research team. Once there was agreement, coders coded the rest of the transcripts; each coded transcript was reviewed by two separate coders to achieve interpretative convergence. Additional codes were added to reflect Latino cultural constructs that did not fit the original precarity framework and emerged inductively in the data analysis. We identified when saturation was achieved by making connections among codes, writing memos, and having iterative discussions. The senior coder is a Latino researcher and was in constant discussions to make sure the coding of cultural traditions was accurate. It was determined that culturally infused themes other than awareness/self-management and limited services pointed to the precarity of participants. These additional themes were rooted in Latino culture. These culturally infused themes captured the context in which these Latino participants experienced living alone with cognitive impairment. The final themes are detailed in the following results section.

## Results

We conducted 123 interviews with 22 Latino older adults living alone with cognitive impairment, the majority of whom (17) did not receive a diagnosis for their cognitive impairment. Most participants were female (*n* = 17), monolingual Spanish speakers (*n* = 16), had high school or less education (*n* = 18), and were born outside the US (*n* = 18). We also interviewed 4 members of participants social circles and 3 Latino providers supporting older adults with cognitive impairment (see Table 1).

The results show that Latino culture permeated and intersected across the lived experiences of participants living alone with cognitive impairment. Precarity was prevalent in all participants’ lives and was exacerbated by familismo combined with cognitive impairment. While emergent themes are consistent with those found in previous research using the precarity framework (Portacolone et al., 2019) and with research on Latino elders reframing the concept of familismo (Ruiz & Ransford, 2012), additional themes beyond the framework reflect cultural factors across the lived experiences of Latino older adults, such as maintaining dignity, resourcefulness, difficulty managing affairs, limited understanding of cognitive impairment, familismo and family conflict. Themes influencing the precarity of Latino older adults living alone were organized through the major areas of the precarity lens; 1) Limited awareness of cognitive impairment; 2) Self-management of cognitive impairment; and 3) Lacking tailored services for cognitive impairment.

### Limited awareness of cognitive impairment

All participants reported a limited understanding of their cognitive impairment. Furthermore, most of them (17) did not report having received a diagnosis for their cognitive impairment. For example, a female participant reflected on her increasing awareness of her limited memory, something she could not ignore as she had in the past:

Yeah, but my memory isn’t as good as it used to be. I don’t know why, I used to say I had a lazy mind. You know? I really, I didn’t tell myself that it was important to keep that. So you know, I thought it was normal to just sort of let it pass. But now, I notice it.

– Woman, Age 91

Some engaged in minimizing their cognitive issues while emphasizing their ability of managing the household alone. For example, a female participant who we observed experiencing significant trouble keeping up the home and taking medications explained:

Because she [my daughter] sees I have a memory problem. But you know what? I’m doing very good in fact. I’m doing very good. Because I do things on my own, you know? I live by myself. So I do everything by myself. Sometimes she will remind me of things, but when she reminds me, I already done it.

–Woman, Age 78

Many families and friends seemed to have difficulty understanding the hardship of living alone with cognitive impairment for their loved ones. For example, adult children often held expectations of their parents that were not realistic given their cognitive impairment, pointing to their limited understanding of their parents’ cognitive health. For example, the son of a participant was frustrated with his father’s hoarding behavior and shared:

In his [my father’s] mind, everything that he has there serves a purpose…I would like to throw away everything that he’ll let me throw away. I imagine that there are even insects in there. I came here on vacation. I’m going to stay with him and put the vacation to the side… Bringing him to my house, I can’t do that. I have my family and I can’t take on that responsibility.

–Son of a Man, Age 81

For this participant’s son it was overwhelming to solve his father’s living conditions while ‘on vacation’ and expected a quick solution to the accumulation of objects for behavior that had been there for years. Further, the son was not prepared to engage in a long-term solution due to family conflicts. After the son’s departure, the participant’s cognitive impairment precarious living situation exacerbated to the point that we reported his case to Adult Protective Services because of the presence of rotten food, increased confusion, and isolation. The participant passed away a few months after the son’s visit, for causes that we could not understand.

### The challenges of familismo

There is an expectation, rooted in cultural values and history, of the adult children and/or women in the family to care for the older adult as well as to live/be close to them. For many of the participants, family conflict arose from gender role-based issues. For example, daughters doing the caregiving and being expected to manage the older adults’ affairs, or the participants having to address their daughters-in-law to get permission to visit their son’s home. For example, this female participant lamented not being able to visit her son:

My son who lives here has never told me ‘Mom, come here’. Why? Because his wife doesn’t get along with me. I’ve tried, but she’s difficult. So I prefer to be far from them.

-Woman, Age 74

Such gender-role based family conflict can lead to the further isolation of older Latinos living alone with cognitive impairment.

In other instances, there was tension due to burdening one sibling with most of the care of their parent with cognitive impairment. The cultural expectation is that all collaborate to divide care among siblings, so there is a kind of breach when others remove themselves from the care. This removal leads to cultural fragility and stress for the caregiver who may not be prepared to confront the familial dynamics. Given the demands of caregiving for a family member with cognitive impairment, this cultural breach exacerbates the already stressful role. In the following quote, the daughter of an older Latina with cognitive decline talks about the lack of engagement from her siblings:

There are eight of us. There are seven others, but I’m the one who fights for her.

- Daughter of a Woman, Age 91

Further, the following quote is from an older Latina living alone with cognitive impairment. She laments how her relatives who live abroad do not serve as support to her, thus contributing to her isolation and presenting conflictive issues.

Yes, because the other granddaughters are in Costa Rica. They went to Costa Rica. They used to call me every month or every two months, but they haven’t called me. The ones who live with my mom only call me to give me trouble. Just to talk to me and ask me for money and say there isn’t money for electricity. I don’t know. They only give me problems. I don’t even like to talk to my daughter-in-law…I don’t even want to talk to her because she just asks me for money. No, if I don’t work, how are you asking me for money?

– Woman, Age 72

The changing social and cultural expectations seemed to transform familismo into a dysfunctional dynamic. For some participants, there was a sense of nostalgia and a sense of loss for their former sense of familismo. In the past, adult children adapted to care for the parent, which contrasted with the new reality, where the older adult accommodates their children’s needs. The following quote illustrates how an older Latina had to adjust from living in Los Angeles, where she felt well and settled to be closer to her daughter.

and then that’s why I’m here, because she decided it’s better that I live here so I’m close to her. So it’s about 40 minutes driving there. But I used to live in L.A.

– Woman, Age 78

Another participant expressed nostalgia for the way familismo was functioning while she was growing up in comparison to her own children.

But everything changes in that sense too, the way they were brought up. Yes. I had my grandmother, my mother, my elder sister who hadn’t married, with me.

– Woman, Age 74

One participant expressed that moving around to be with her children and their families who lived in very distant locations within the United States was similar to taking a vacation. For those living with cognitive impairment, adjusting to constant moving can be difficult.

But I’ve been very close to my children, so yeah. So if the wives want, if the wives want it, they’re very nice wives really. So we’ll see. but he has a very nice, sweet wife, very nice wife too. So yeah, they’re all really quite nice, the girls. So if they’re willing to have me, I call it like a vacation.

– Woman, Age 91

From some of the older adult’s perspective, this new reality has left them displaced and more alone.

Yes. So my house was always full. So, I - I thought that because I had many people - and now I’m alone. In the past I wanted to cry. Now I don’t. Now I can talk about it.

– Woman, Age 74

Yes. My son had been in jail, and he got out of jail and went to Tijuana. Then, he came back, and he didn’t want to come back here, so we went to Las Vegas. We stayed in Las Vegas for many years… he came back here and left me there.

– Woman, Age 80

### The impact of migration on living alone with cognitive impairment

Resettling into new places after cognitive impairment can be very challenging and confusing because the older adult is being removed from what is familiar and can contribute to disruptions in services. Compounding the issue is how families are spread across the country and world, which may shape the extent to which older adults are rooted in their environments when moving to be in proximity to their children and families. The following quote is from an older Latina living alone with cognitive impairment and feeling unsettled because her daughter feels she will be better off in her country of origin, but she feels it is more difficult to manage health and finances there.

Yes. That’s why my daughter tells me, ‘Mom, go to Nicaragua. You’ll be with your whole family’. But I don’t want to go. I don’t want to go to Nicaragua. And there, there aren’t medications. The medication is very expensive. I also don’t have anything to live off of.

– Woman, Age 72

Another participant living alone with cognitive impairment shared having to make changes and lose freedom because of older age.

When you’re young, you are free to go wherever you go, because you can move around. But when you’re an elderly person, you can’t.

– Woman, Age 74

Others felt they were misplaced in the U.S. and longed to go back to their place of origin due to climate and culture. There was resentment to the decision to be in the U.S. at the insistence of their family who determined that they were better off nearby and not being alone.

And that’s why I would like to go to Guadalajara instead. Because there, it’s different. The climate there is good. I didn’t feel too hot or cold there. When I came here as a tourist, I could only handle being here a month because everything hurt. I left and I was fine. It’s that - and I can’t handle this climate here…When we came, they were saying, ‘Come, come’. I didn’t need to come here. I had money there and everything. I had a house and everything. But no, ‘Come here so you’re not alone’.

– Man, Age 94

### Family conflict within Latino culture and caregiving for older adults with cognitive impairment

While late life has historically been a time of Latino elders being cared for by younger family members, westernization of cultures and globalization of individualism have impacted the traditional expression of familismo. Many of the family conflicts described by participants seemed to be rooted in expectations of familismo. For example, the framework of aging cast by familismo suggests that the older adult must be cared for by family or at least live close by. In an effort to hold to this value, some older Latinos were relocated by family members, despite the older adult’s own wishes and what may have been best for them given the stress of relocation on cognitive impairment. Some participants expressed passivity in allowing their family members to determine where they would live. For some participants, the decision was based on family structure and the gender of the main caregiver. Yet, there was dissatisfaction with the uncertainty and the changes that could be exacerbated by their compromised mental state brought about by their cognitive impairment.

I moved here because of my daughter. And my son’s still over there, but he has a family and a wife, so the decision was for me to come here…

– Woman, Age 91

After she moved, to relieve the daughter, the adult children all living 3 different US states agreed that their mother would have lived for 3 months with each one of them. The participant explained:

And you know, then they talk about me going to spend three months with everybody. It is kind of, I wonder well gee [slang], do I want to do that? Do I want to travel?

– Woman, Age 91

For others, family did not fulfill their expectation of familismo as they did with their own parents.

My difference is my mother, when I was married, she always lived with me. So maybe somehow I thought the same would happen with my children. But it wasn’t, because children are different. but each of them has their own life. That’s why there are many women who are alone. I don’t want to think much about it, because as I say, it hurts your heart, your feelings. And no more. I say, ‘Lord, Holy Father…’

– Woman, Age 74

Some also found themselves isolated after a lifetime of building relationships.

In the past, it hurt. I thought ‘To think that I had so many people and now I’m all alone’. And I say, it’s possible that the same happens to all the ladies. So I thought that I might have that too. ‘But well’, I said, ‘perhaps I don’t deserve it, so it’s okay’.

– Woman, Age 74

Many participants expressed a disjunctive with resettling into new places after cognitive impairment and how the geographic dispersion of families may create disruptions in the older adults’ environments when moving near adult children to receive care. Some participants expressed going without what they needed or pretending they do not have needs so that their children would live comfortably or not worry.

so you took these very big decisions and painful decisions to leave a place that you really, really loved to make them feel relieved.

– Woman, Age 91

I don’t remember how long. It was a while before I came here, because my daughter brought me. If not, I wouldn’t be here. I would be there. I like it better there than here. But she sent for me so that she could have me here close by.

– Woman, Age 94

However, many times there was also a melancholy tone tied to the cultural expectation of living and being cared for by family during late years as depicted in the traditional framework of familismo (Ruiz & Ransford, 2012). While the family had been getting involved with her care, geographic dispersion was becoming overwhelming for one participant with cognitive impairment.Yet she went along because it was time she got to spend with her children:

Like right now they’re talking about maybe my spending some time with them in the summertime. But my daughter-in-law in Florida, she doesn’t work, so I can go just about any time, I have someone there. And then, what’s the other one that I have? Oh, my son and my daughter-in-law in Illinois, they both teach, so I’d have to go sometime in the summer or in vacation time. So it’s whatever I want to. I don’t have to. You know, [in] a year or two, when you get older I think it’d be a pain in the neck…I say right now it’s fine, but maybe when you get older it would be kind of a pain, kind of a nuisance to be picking up and moving around so much.

– Woman, Age 91

### Self-management of cognitive impairment

All participants employed a range of resources that seemed to support their ability to manage the precarity of living alone with cognitive impairment. Some adaptations centered on independence while others may be collectivistic in nature, hence more reflective of the Latino culture. Collective experiences included attending religious services and expanding the definition of family to include caretakers as kin, as a means to cope with the fact that their children were not really there/sporadically present. In many cases, the resourcefulness that participants found to manage life was exceptional. For example, all participants went to great lengths to accomplish things even when they were on their own, but these tasks may have been much less difficult if they had support. One participant described her experience of feeling unwell and deciding to take public transportation to the hospital instead of reaching out to family or calling an ambulance for help. Her narrative highlights the depths of inner resources and resiliency many participants called upon

I walk with a horrible headache, sit on the bus and then wait and get down, and then I have horrible headache and they put me in a room, and they told me in the room, you had an aneurism, and that’s it. But I stayed there for two months I was in recovery.

– Woman, Age 78

One participant carried a seat with her when she walked so that she could rest and continue attending a senior center, something that brought her joy.

The cartilage is gone in my knees. Look at how they swell up. They hurt so much because bone is hitting on bone. Walking causes a lot of pain. But no, I walk like this because I have to go…Sometimes… look. I have my little stool there. Sometimes I walk from here to my house on 17^th^ but with rest. I walk a block, and then I sit down. I bring it with me.

– Woman, Age 65

With a similar level of determination, all participants also took great lengths to seek the connections they needed as well. Attending church, community centers, gatherings, and even just walking with friends were done with resourcefulness. A participant accepted the help of a home health aide to get to the community center despite not wanting the support, she valued the connectedness at the center more than being alone and used her resources to get her needs met. One participant traveled for several hours to get to church each Sunday because the service was in Spanish. He explains the value as:

[I spend there] All day, because I leave here at 9:30. I have to take a car from here…From [there] I have to take another car that brings me to the river. Where the castle is. I arrive at 10:30, and at 11 the prayer begins. After, we go to the second part, which is classes. In the classes, it’s about reading the passages of the evangelist. That’s one hour. Then, we go to another meeting which is…priesthood, they call it, where they give a lot of talks and expositions about taking on the priesthood… That is for two hours…Five hours, and from there they present the baptism. They baptize people, so you leave at six in the evening…[to return] I have to wait for the car. It depends if a car passes, or the bus. I get here in an hour, more or less.

– Man, Age 81

### Desire for autonomy

Autonomy was important because it was associated with dignity. Most older adults associated autonomy with the maintenance of dignity. For example, taking pride in completion of basic activities like bathing and cooking, and being able to meet one’s own needs through modification rather than seeking help. Many participants articulated a forced tension between desire for autonomy and the desire for connection, in which dignity seems paramount in order to survive. In the context of familismo, dignity is also about managing disconnection with family, so having to do things alone represents a necessity but not a cultural preference.

…thank God I feel like I’m healthy and I do a lot of things on my own. I take my shower, I do my food. And then when I get bored, I go for a walk. Most of the time by myself. And this is the story of my life. *But you have to do it*. When you live alone you have to learn to do things, you know what I mean?

– Woman, Age 78

While some older Latinos expressed a forced tension between autonomy and surviving, others seemed to approach the loss of independence with grace, which is a Latino cultural practice to maintain dignity. A participant explains her perspective on her loss of independence

So it’s a time, it’s a time. That’s it. You like to be independent, but you know, there’s a time when it just, you got to accept that something’s going to happen, and you can’t do anything about it.

– Woman, Age 91

### Maintaining dignity

Many participants desired to live autonomously, make their own decisions and direct their own lives. Maintenance of dignity by not burdening their filial and family networks in late life for older Latinos with cognitive impairment living alone is influenced by cultural expectations of familismo as well as the desire for independence and personal identity. Some participants had difficulty with maintaining dignity in not bothering their children, which at times interfered with getting the services they needed:

I live alone because of my children…. it’s difficult having an elderly person…they live far away… ‘No, I can’t be a burden for him. I’ll work. I’ll work’.

– Woman, Age 74

This quote articulates yet another notion of why she prefers to remain in the United States despite much of her family being abroad. The participant explained she does not want to be dependent on her children:

My son in Puerto Rico says Mom come to live here if you want…but it’s hard…they also have to take me around, but here instead I manage myself. That’s the advantage.

– Woman, Age 74

### Reframing

Most participants also used psychological resources to cope with precarity. The skill of reframing, finding alternate ways of interpreting a situation, was prevalent among participants. Reframing and focusing on what is within one’s control was also prevalent for most participants in fostering acceptance of their situations with much resignation:

Oh God, that’s a good one. I think anything that overwhelms you, it really isn’t significant, that you’re really too old to let that bother you, really. But you have to know when to decide that. When to accept it. But you know, I just, I look at life, a lot of it, I don’t have much time on this earth so I’m not going to let anything bother me.

– Woman, Age 91

When faced with difficulty that threatened to shift or upend one’s identity or cause suffering, older Latinos used religion and acceptance to support shifting their perspectives and reframing thoughts. When considering living alone, despite her preference to be surrounded by her children, one participant relied on her faith:

…The bible says you’ll leave your father and mother that’s the most correct thing, God planted when they get married.

– Woman, Age 74

In this way, it seems that for this moment the participant was able to place her loneliness within a religious context and find a sense of purpose for her isolation. That is, in denying her own desires, she holds that she is allowing her children to act in a way that is according to God’s will.

### Avoiding being a burden

Living alone with cognitive impairment is complex. For many older Latino’s living alone is not culturally normative. Yet, some participants displayed a determination to maintain their independence and avoid being perceived as a burden on family members and friends despite the difficulties they had meeting their own needs, loneliness, and disappointment in their living situations. They often chose precarity over feeling like a burden.

Researcher: You told her about [what] happened to you, that you were sitting on the sofa for three days?

No. No, I didn’t tell her [daughter?]. I didn’t tell her anything, because I don’t like to worry her. She realized because when she came in August, I couldn’t walk. I couldn’t get out of bed. She realized. I couldn’t walk for three days.

– Woman, Age 72

### Lacking tailored services for cognitive impairment

Several participants navigated limited access to much needed services including culturally and linguistically appropriate services (CLAS), support with ADLs, transportation, and insurance. The causes of the lapses in access are varied but many related to migration, immigration status, lack of awareness of cognitive impairment, and frequent relocation shaped participants experiences gaining access to services. The hardship of supporting older Latinos living alone with cognitive impairment was tangible when a Latino physician who we interviewed burst to tears when asked about his experience. He explained:

This is one of the most difficult places to be as a medical provider… One of the most difficult. It’s hard not to feel that vulnerability. So you want to be in a space where you are able to hear what they’re experiencing, and you know that there’s a whole world that they’re not sharing with you.

– Latino Medical Provider, Age 56

Compounded with the limited information from patients, he highlighted the difficulty of having ‘limitations of what you have to offer, and you know that they’re going home with some measure of some suffering that you’re not likely to impact. it just mounts and mounts and mounts. That’s a challenging place. It’s one of the more challenging’. – Latino Medical Provider, Age 56

Participants further expanded on the challenges of receiving services. For example, a female participant pointed out that many services at a local senior center have been taken away over time. Despite the demand and complaints from Latinos. She explained:

The bus that they had before was taken away. They’re complaining about all of that. Before, there were English classes and classes for citizenship, computer classes. They took away all of the services and a lot of people are complaining about it.

– Man, Age Unknown

Some participants had difficulty navigating the immigration process and needed services (e.g. health insurance or SSI) were difficult to access due to immigration statutes, language barriers or cognitive impairment. A male participant expressed fear about seeking assistance because of his immigration status and the possibility of being detained:

I retire from work, they’ll pay me 400 dollars a month, and to go and claim it. They’ve told me that I should go to immigration to claim my green card. I’ve talked with people, and they’ve told me, ‘don’t go’. Because it’s dangerous there. If you don’t have all of your documents in order, they detain you there.

– Man, Age 81

### Limited access and awareness of services

Most participants did not have access to services they needed to complete basic ADLs and to maintain their lives; including insurance, support in the home, access to CLAS in healthcare and housing. Further, many participants were confused about how to access services. We asked participants about their knowledge of transportation services; this is a quote from a wheelchair using diabetic Latino man living alone with cognitive impairment.

Researcher: The next one is Para Transit, and it’s a van or taxi that can come to pick you up to bring you to your appointments or to go shopping. Have you heard of this program?

No, but I see that other people use it. I see it, but I haven’t used it. I haven’t asked for it. I don’t know how they did it. I don’t know, but it was there…Yes, it’s very necessary because to go to the hospital I have to take two cars. I have to wait for one to go past. Then, after that journey it’s another to go to the other place. Another period of waiting for another car to pass by. There are times when they leave you far away. Yes. I would use that assistance

– Man, Age 81

In some instances, participants described difficulty navigating the healthcare system. As this male participant relates explains, not finding help would come at the expense of his wellbeing, thus, resulting in confusion and helplessness.

Yes. The only thing that I can tell you is that I just went to the clinic, because my machine for checking my blood sugar doesn’t work. It has been a week, and it doesn’t work. I should check myself in the morning and at night. I don’t know what’s wrong with it…because from the second floor, they send me to the third. From the third, to the second. No. I don’t know what’s happening, and I just came home instead. I don’t like it.

– Man, Age 69

Difficulty managing affairs was common among most participants. Many participants had difficulty managing basic aspects of their lives including finances, shopping, and navigating services to get their needs met. For example, for some participants, routine tasks such as paying bills were hampered by memory loss, but also by literacy and the ability to read in English. A researcher noted limitations with resolving financial difficulties with one of the male participants due to language and immigration status.

One issue seems to be some bill collectors and agencies sending information only in English. Also, fears about being deported keep him perhaps in poverty and some isolation too. I wonder what he would do differently if he had the green card?

– Research Note from Interviewer

The male participant who we reported to Adult Protective Services because of his limited supports was concerned about completing the requirements for U.S. citizenship due to the language and memory issues. Yet, citizenship would allow him to obtain basic needed services to help manage living alone with cognitive impairment but getting to that goal with cognitive impairment was like a maze. In addition, because of his cognitive impairment, it was extremely difficult for him to follow the steps to get the green card. For example, when a researcher accompanied him to immigration services, the clerk explained to the researcher that he had already been to their offices several times and that she had explained the processes to him—his cognitive impairment prevented him from retaining this information, thus returning to seek services he had already accessed. He explained:

I don’t know how I’m going to get my citizenship, because I can’t learn the questions. I don’t retain them. I don’t know…in the mind…I could never remember the lessons at school. That’s why I didn’t study.

– Woman, Age 72

## Discussion

This study aimed to examine cultural expectations and experiences of Latino older adults’ concerns and priorities about living alone with cognitive impairment through the lens of the precarity framework. The results present that those Latino older adults living with cognitive impairment in the San Francisco Bay Area experience deep difficulties in most aspects of their daily life and these difficulties are layered within their cultural context, which in most cases had an aggravating effect. The role of younger family members in the lives of older Latinos is shifting (Ruiz & Ransford, 2012). For these Latino older adults, culture and family did not play a protective role. Using precarity as a framework proposes that coping with cognitive impairment while living alone exacerbates uncertainty, cumulative pressures, desire to remain independent, and limited access to appropriate services in three key areas: 1) Limited awareness of cognitive impairment; 2) Self-management of cognitive impairment; and 3) Lacking tailored services for cognitive impairment. The findings in this study showed these key areas of precarity were present in the experiences the participants shared as well as in the observations made by the researchers. Further, the participant narratives extend the precarity framework to reveal how the Latino culture intersects with the experience of precarity while living alone with cognitive impairment, especially in reference to the role that family plays through the expectation of familismo.

Similar to previous findings on White populations, older Latinos living alone in this study experienced health threats including self-neglect (Gibson & Richardson, 2017; Meaney et al., 2005; Miranda-Castillo et al., 2010; Tierney, 2007), malnutrition (Miranda-Castillo et al., 2010; Tuokko et al., 1999) and falls (Miranda-Castillo et al., 2010; Tierney, 2007; Tuokko et al., 1999). Further, older Latinos in this study experienced difficulties in cognitive impairment management directly related to cultural experiences, this finding supports the notion that culture may have a nuanced impact on cognitive impairment among Latinos (Perales-Puchalt et al., 2021; Rote et al., 2019; Ruiz & Ransford, 2012) especially those who are not born in the US (Gentry et al., 2021). The participants in this study are largely immigrants who left their home countries for work in the United States often sacrificing their education to do so. Most participants had no college education which may account for some of the difficulty understanding cognitive impairment. However, there seemed to be avoidance of acknowledging the consequences of cognitive impairment while living alone. Such avoidance is reflected in minimizing the symptoms of cognitive impairment combined with their living conditions and the lack of supports. Although living alone may not have been their preference or cultural expectation, the perceived options, including family and services available were extremely limited. Further, family members were also limited in understanding essential needs that their relatives with cognitive impairment were experiencing to the level of precarity. In the context of migration, resources diminish due to the demands of maintaining employment and adult children taking care of their own separate households. In some cases, this resulted in unreasonable expectations of how much their parents with cognitive impairment could really do on their own. While in other cases, there was little to no involvement at all. Even when there were families with multiple siblings, not all the adult children were actively involved in the care coordination of the older adult with cognitive impairment, and when there was coordination, the solutions were more burdensome to the older adults, such as traveling around to be with different family members. Such detachment patterns are not part of the traditional Latino culture for older generations, but it may be becoming more prevalent with newer generations due to work and life demands within United States. Thus, the reliance on family that is alluded to Latino culture may not be as present in migration contexts.

While family patterns in the traditional Latino culture are not rooted in independence as a marker of well-being, older Latino participants expressed a desire to not be a burden. In the context of Latino culture, the results indicated that this desire to avoid being a burden seemed to be tied to feeling they had to be independent. Thus, their self-management of cognitive impairment may be a means of protecting adult children from burdening themselves and disrupting their own daily patterns. Conversely, none of the participants shared that they felt anyone confided or depended on them. This one-way relationship may be contributing to isolation and further feeling like a burden, which is counter to the cultural expectation of a collectivist culture.

Many participants resorted to reframing loneliness as independence with statements that affirmed their identities in relation to family and friends as well. Some of the reframing was rooted in religion as a source of strength and support for their situation. Others rooted themselves in praising the success of their children to explain their lack of involvement and support. Nonetheless, we theorize that this reframe provides a sense of identity continuity which may be stabilizing and consequently contribute to coping with precarity.

The third major area for the precarity framework is lacking appropriate services for cognitive impairment. Participants reported having many and complex difficulties accessing services. While some adult children were managing services for their parents, it was difficult for the adult children to know how to assess their relative’s need for such services. Further, several participants were left to their own devices with unreliable services and in some cases no services at all. While social services representatives seemed to be tangentially involved in monitoring the well-being of the older adults, there seemed to be a lack of access and continuity to assessment outcomes and follow-up. Without formal service professionals to provide guidance and/or referrals to resources, the onus of keeping informed is on the communication between the older adults living alone with cognitive impairment and their families. As a result of cognitive impairment and cultural dynamics, there was often a gap in communication from the part of the participants with their families and/or other points of contact for seeking help. Further, migration as well as immigration status presented a barrier to seeking or accept services. As participants shared, migration resulted in displacement of older adults from a familiar setting to an unfamiliar one. This displacement includes lack of knowledge about the agencies, services, and other systems that create uncertainty for older adults living alone with cognitive impairment. For the participants in this study, the catalyst for migration was being closer to adult children, yet those children remained limited in their capacity or will to actively engage in caregiving coordination. Consequently, the older Latinos found themselves having to adjust to living alone with cognitive impairment in an unfamiliar environment. Many times, participants relied on their own resourcefulness to obtain basic needs, but at a high burden to their safety and health.

In addition to the uncertainty caused by migration into a new community and culture, immigration status was yet another complicating factor. One of the main consequences of immigration status was isolation due to distrust. For many participants trust was at the center and often in the way of obtaining services. Even when family members were not available to assist them, participants communicated discomfort with outside care providers, sometimes considering them dangerous due to fear of deportation. In the few cases that there was acceptance of an outside caregiver, they came referred from trusted sources. Once trust was established, outside caregivers tended to take on the role of family, given the gaps in personal attention from their own family members. While in cases when there was no outside care, the participants might go to senior centers and churches to fulfill the need for companionship. In a migration context, familismo still proved applicable, yet the construct needs further refinement in relation to the capacity of family to be effective caregivers. Cultural expectations may also prevent that participants disclose the full extent of their needs to preserve dignity and avoid being outside of the collective cultural mold.

## Limitations

There are some limitations to this study that need to be highlighted. Although this article examined the various experiences of older Latinos living alone with cognitive impairment, we did not capture the full experiences of their families. However, we did collect data from the family members with whom they interacted most. Another limitation was that given the diversity found in the Latino population, the sample of this study cannot be generalized to the entire Latino community because there are many subgroups of Latinos not included in this study. Further, the use of qualitative methods limits the findings to the perception and context of the older Latinos in the study.

## Conclusion

The three major areas of the precarity framework were very useful to examine the lived experiences of older Latino adults living alone with cognitive impairment. Markers of the precarity framework were present in the participant narratives around self-management of cognitive impairment. In addition, participants’ shared experiences in the context of the Latino culture were a cause for socio-cultural precarity. Within the context of migration, traditional cultural norms and practices may not be applicable due to work demands and generational cultural differences. Familismo played an important role in the efforts of families to maintain a sense of dignity and functioning at the same time. However, given the difficult demands of employment and raising their own families, familismo can become more like an ideal than a practice. Further research is needed to better understand how to bridge the gap between the needs of these older Latino adults living alone with cognitive impairment, their families, and formal services.

## Figures and Tables

**Table 1.** Participant characteristics.

Older adults living alone participants’ characteristics (*N* = 22)	
	*N*
Age (mean, range)	83 Mean years (SD = 10.92)
	(range 65–103)
Cognitive impairment category	
MCI, n	3
Alzheimer’s disease, n	2
MoCA ≤ 24, n	17
Gender female (%)	16 (76%)
High school or less education (%)	18 (86%)
Monolingual Spanish Speakers (%)	16 (76%)
Foreign born (%)	18 (86%)
Informants participants’ characteristics (*N* = 4)	
	*N*
Age (mean, range)	50 Mean years (SD= 10.07)
	(range 41–61)
Gender female (%)	3 (75%)
High school or less education (%)	1 (25%)
Monolingual Spanish Speakers (%)	3 (75%)
Foreign born (%)	3 (75%)
Providers participants’ characteristics (*N* = 3)	
	*N*
Age (mean, range)	57 Mean years (SD = 2.83)
	55–59 (range)
Type of provider	
Physician	2 (67%)
Unknown	1 (33%)
Providers, ethnicity	
Hispanic/Latino	3 (100%)
Gender female (%)	2(67%)
High school or less education (%)	1 (33%)
Monolingual Spanish speakers (%)	0 (%)
Foreign born (%)	1 (33%)
